# miR-26a exerts broad-spectrum antiviral effects via the enhancement of RIG-I-mediated type I interferon response by targeting USP15

**DOI:** 10.1128/spectrum.03124-23

**Published:** 2023-11-29

**Authors:** Jikai Zhang, Chunyang Li, Yao Hou, Dan Liu, Qiudi Li, Zijie Wang, Renxian Tang, Kuiyang Zheng, Hongbo Guo, Wenshi Wang

**Affiliations:** 1 Department of Pathogen Biology and Immunology, Jiangsu Key Laboratory of Immunity and Metabolism, Xuzhou Medical University, Xuzhou, China; 2 Jiangsu International Laboratory of Immunity and Metabolism, Xuzhou Medical University, Xuzhou, China; Regional Centre for Biotechnology, Faridabad, Haryana, India

**Keywords:** innate immunity, miR-26a, USP15, RIG-I, ubiquitination, broad spectrum

## Abstract

Host innate immunity is an important defense line against virus infection and is precisely regulated by various factors. Studying the mechanism of virus-host interaction is essential to developing novel antivirals. Being one of the key host natural antiviral responses, host small non-coding RNAs (miRNAs) possess promising antiviral potential worthy of study. Herein, we found that miR-26a exerted broad-spectrum antiviral effect against multiple viruses, e.g., Hepatitis E virus, Vesicular Stomatitis Virus, and Sendai Virus. Mechanistically, miR-26a specifically targets 3′UTR of mRNA to inhibit USP15 expression. USP15 interacted directly with RIG-I to deubiquitinate K63-linked RIG-I, thus negatively regulating type I interferon (IFN) signaling. Consequently, miR-26a, by downregulating USP15, promotes K63 ubiquitination of RIG-I to enhance type I IFN responses, resulting in an active antiviral state against virus infection. Intriguingly, the activation of type I IFN responses could suppress miR-26a expression, serving as an intrinsic negative feedback loop to avoid dysregulated signal activation. Hence, the broad-spectrum antiviral effects of miR-26a and its mode-of-action enriched the interaction networks between miRNAs and innate immunity, providing insights for the development of broad-spectrum antivirals against viral infection.

**IMPORTANCE:**

miR-26a serves as a potent positive regulator of type I interferon (IFN) responses. By inhibiting USP15 expression, miR-26a promotes RIG-I K63-ubiquitination to enhance type I IFN responses, resulting in an active antiviral state against viruses. Being an intricate regulatory network, the activation of type I IFN responses could in turn suppress miR-26a expression to avoid the disordered activation that might result in the so-called “type I interferonopathy.” The knowledge gained would be essential for the development of novel antiviral strategies against viral infection.

## INTRODUCTION

Innate immunity serves as the first host defense line against viral invasion and infection. Pathogen recognition receptors (PRRs) recognize viral DNA or RNA and trigger the innate immune responses during viral infection. RIG-I-like receptors (RLRs) are major PRRs for RNA virus-triggered antiviral response. Especially, retinoic acid-inducible gene-I protein (RIG-I) and melanoma differentiation-associated protein 5 (MDA5) are regarded as vital RLR family members for the recognition of viral RNA (1). RIG-I or MDA5 can recruit downstream adaptor mitochondrial antiviral-signaling protein (MAVS) and subsequently transduce signals through TANK-binding kinase 1 (TBK1), thereby activating interferon (IFN) regulatory factor 3 (IRF3) to induce type I IFN production (IFN-α and IFN-β) (2, 3). After binding with type I IFN receptors IFNAR1/2, JAK-STAT signaling cascade was subsequently activated; finally, more than 300 interferon-stimulated genes (ISGs) are induced to restrict virus replication (4, 5).

Small non-coding RNA sequences (~23 nt), known as microRNAs (miRNAs), possess huge antiviral potential worthy of study (6). Being one of the key natural antiviral responses against RNA viruses, host cells produce more than 2,000 miRNAs to regulate over 60% of all human protein-coding genes. Upon viral invasion, miRNAs bind to key host factors to elicit robust antiviral innate immune responses. For example, miR-24, miR-124, and miR-744 were shown to downregulate the p38 mitogen-activated protein kinase (MAPK) signaling pathway, promoting the production of cytokines and resulting in a broad-spectrum antiviral state (7, 8). Conversely, viruses have evolved to harness miRNAs to suppress key antiviral pathways, creating a virus-friendly microenvironment to facilitate replication. For instance, Epstein-Barr virus upregulates miR-155 to suppress NF-κB innate immune responses, thus supporting its infection (9). Moreover, miRNAs also interact directly with the viral genome to regulate viral infection. For instance, hepatitis C virus employs miR-122 to stabilize its genomic RNA and promote viral replication (10). On the contrary, miR-28-5p, miR-150, miR-223, and miR-382 have been found to repress RNA translation of HIV-1 via direct binding (11). Therefore, miRNAs play critical roles in intricate host-virus interaction networks by targeting viral genes or host genes. The knowledge regarding the specific roles of miRNAs would be essential for the development of novel antivirals, especially for the targets that are currently not druggable by small molecules.

Previous studies reported that miR-26a could inhibit porcine reproductive and respiratory syndrome virus replication by activating type I interferon response (12, 13). However, the exact mechanism remains elusive. RIG-I signaling could be regulated by ubiquitination modification (14, 15). The Lys 63-linked (K63-linked) polyubiquitination of RIG-I protein is required for its downstream signal activation and transduction. Several studies have reported that some E3 ubiquitin ligase, TRIM25, Riplet, and MEX3C were required for the activation and polyubiquitination of RIG-I (16 – 18).

In this study, we found that miR-26a exerted broad-spectrum antiviral effect against multiple viruses, e.g., Hepatitis E virus (HEV), Vesicular Stomatitis Virus (VSV), and Sendai Virus (SeV). Mechanistically, miR-26a specifically targets 3′UTR of mRNA to inhibit USP15 expression. USP15 interacted directly with RIG-I to deubiquitinate K63-linked RIG-I, thus negatively regulating type I IFN signaling. Consequently, miR-26a, by downregulating USP15, promotes K63 ubiquitination of RIG-I to enhance type I IFN responses, resulting in an active antiviral state against virus infection. The knowledge gained enriched the interaction networks between miRNAs and innate immunity, which thus would be instructive for the development of broad-spectrum antivirals against viral infection.

## RESULTS

### miR-26a exerts broad antiviral effect against multiple viruses

**Fig 1:**
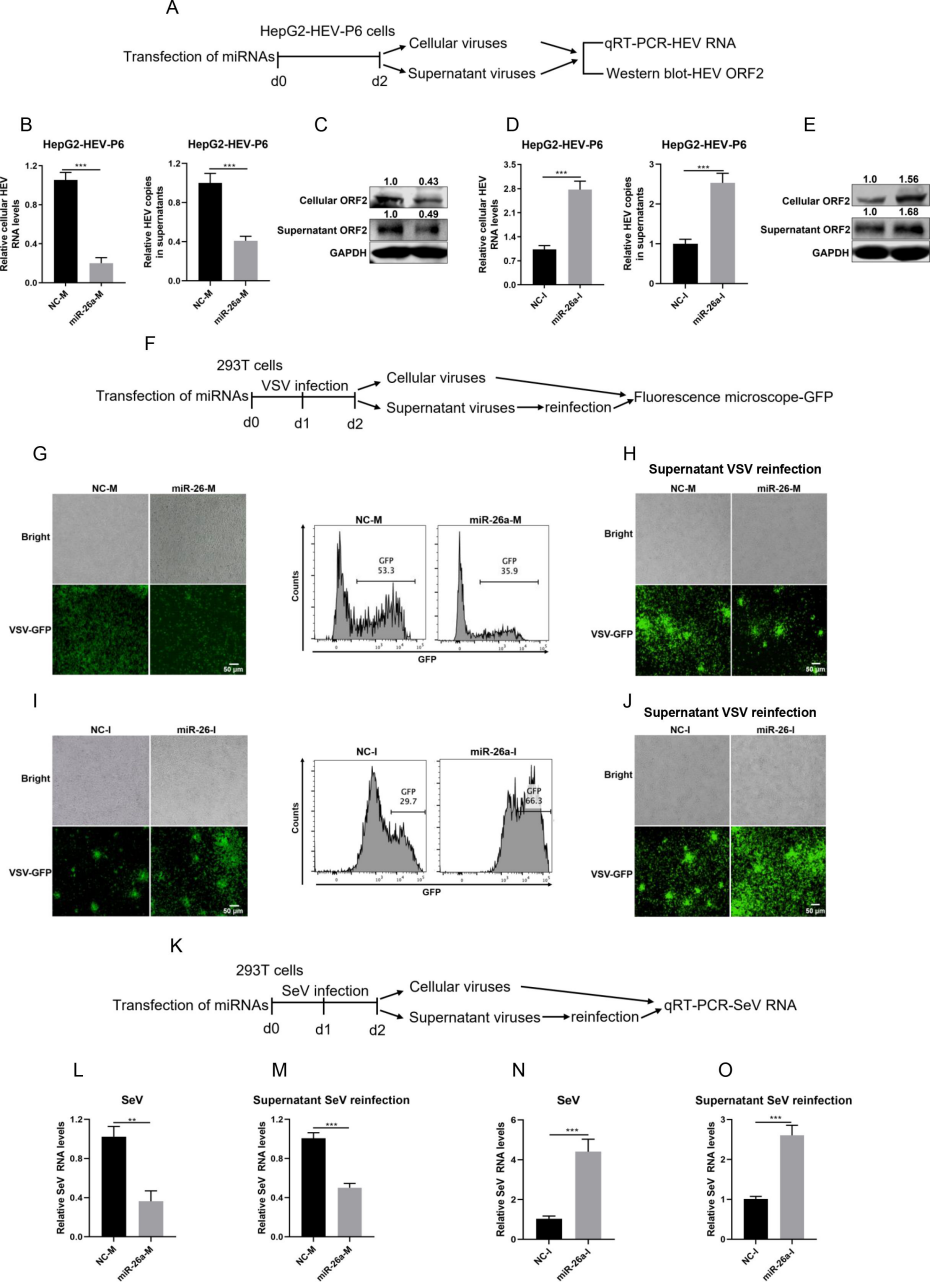
miR-26a inhibits HEV, VSV, and SeV replication. (**A**) The experimental procedures to verify the effects of miR-26a on HEV replication were illustrated. (**B and C**) The NC-M or miR-26a-M were transfected into HepG2 cell-based HEV infectious cell model (HepG2-HEV-P6). The relative levels of cellular HEV RNA or HEV RNA in supernatants were determined by quantitative reverse transcription PCR (qRT-PCR) based on two independent assays with three repeats each (**B**). The cellular and supernatant HEV ORF2 protein was detected and quantified by western blot (WB) (**C**). (**D and E**) Same as **B and C** for the transfection of NC-I, miR-26a-I. (**F**) The experimental procedures to verify the effects of miR-26a on VSV replication were illustrated. (**G and H**) NC-M or miR-26a-M were transfected into HEK293T cells for 24 h, followed by VSV expressing green fluorescent protein (VSV-GFP) infection (MOI = 0.1) for another 24 h. The levels of VSV replication were detected by immunofluorescence microscope analysis or quantified by flow cytometry analysis of GFP fluorescence intensity (**G**). Then, the supernatant was collected to re-infect HEK293T cells for 24 h. The levels of VSV replication were detected by immunofluorescence microscope analysis (**H**). (**I and J**) Same as **G and H** for the transfection of NC-I or miR-26a-I. (**K**) The experimental procedures to verify the effects of miR-26a on SeV replication were illustrated. (**L and M**) NC-M or miR-26a-M were transfected into HEK293T cells for 24 h, followed by SeV infection (100 HAU/mL) for 24 h. The relative levels of SeV RNA were determined by qRT-PCR based on two independent assays with three repeats each (**L**). Then, the supernatants were collected to re-infect HEK293T cells. The relative SeV RNA levels were also determined by qRT-PCR based on two independent assays with three repeats each (**M**). (**N and O**) Same as **L and M** for the transfection of NC-I or miR-26a-I. Data were shown as means ± SEM. Statistical analysis was performed using two-tailed Student’s t test. **P < 0.01 and ***P < 0.001. MOI: multiplicity of infection; HAU: hemagglutinating units.

In order to explore potential miRNAs that intimately interact with RNA viruses, the expression levels of a panel of miRNAs were detected with or without the presence of HEV, a single-stranded RNA (ssRNA) virus (Fig. S1A). Interestingly, albeit most of the miRNAs remain unchanged, HEV decreased miR-26a expression significantly (Fig. S1B), even at multiple time points post-infection (Fig. S1C). Similarly, two other ssRNA viruses, VSV and SeV, also downregulated miR-26a expression (Fig. S1D and E). These results prompted us to check whether miR-26a could regulate viral replication in turn. As shown in Fig. S2, transfection of miR-26a mimics (miR-26a-M) significantly upregulated the endogenous miR-26a expression level, while miR-26a inhibitors (miR-26a-I) markedly downregulated the miR-26a expression level (Fig. S2). Firstly, we assessed the effects of miR-26a on HEV replication according to the experimental procedures shown in Fig. 1A. Indeed, transfection of miR-26a mimics into HepG2-HEV-P6 cells robustly decreased the HEV RNA levels both intracellularly and extracellularly (Fig. 1B). Conversely, miR-26a inhibitors significantly promoted HEV replication and viral production (Fig. 1D). Moreover, miR-26a also markedly decreased the expression levels of ORF2 protein both in cell and in supernatant (Fig. 1C). In contrast, miR-26a inhibitors increased ORF2 protein expression levels (Fig. 1E). Then, we also assessed the effects of miR-26a on VSV replication according to the experimental procedures shown in Fig. 1F. Similarly, miR-26a potently inhibited cellular VSV replication (Fig. 1G), whereas its inhibitor facilitated the cellular viral replication (Fig. 1I). Moreover, the infectious VSV particles produced in the supernatants were also decreased by miR-26a treatment (Fig. 1H), while knockdown of endogenous miR-26a promoted the production of VSV particles (Fig. 1J). Furthermore, the effects of miR-26a on SeV replication were also verified according to the experimental procedures shown in Fig. 1K. Similar effects were also observed in the setting of SeV infection (Fig. 1L through O). In contrast, no significant differences were observed in control cells without transfection as well as cells transfected with negative control mimics (NC-M) or inhibitors (NC-I) (Fig. S3). Therefore, we conclude that miR-26a exerts broad antiviral effect against multiple ssRNA viruses.

### miR-26a potently enhances type I IFN responses to exert its antiviral effect

**Fig 2:**
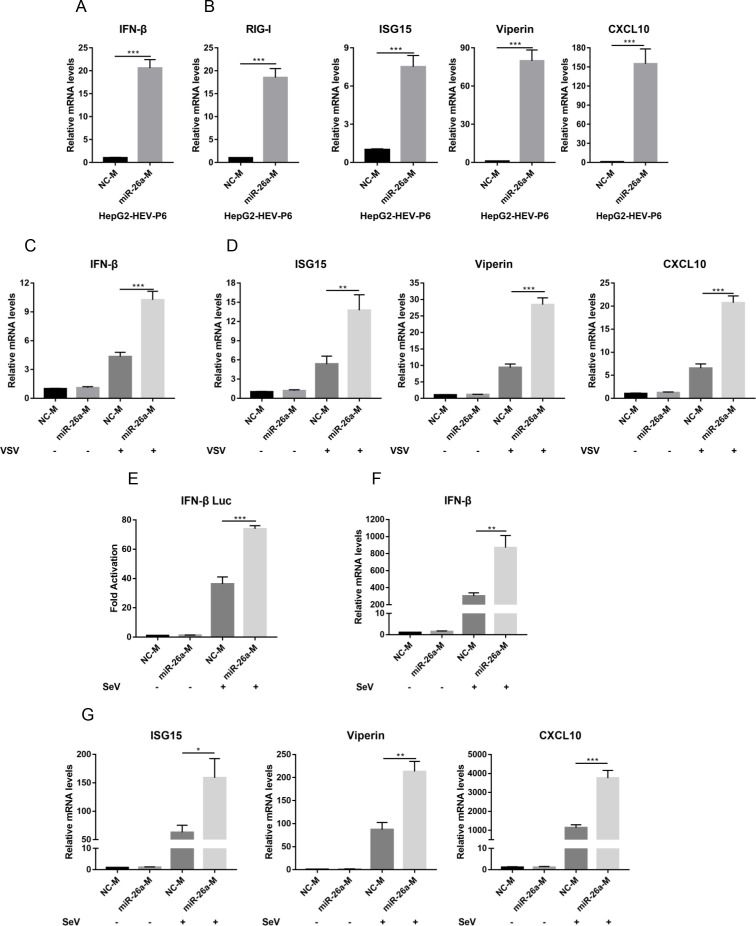
miR-26a potently enhances type I IFN responses to exert its antiviral effect. (**A and B**) NC-M or miR-26a-M were transfected into HepG2-HEV-P6 cells. The relative mRNA levels of IFN-β (**A**) and ISGs (RIG-I, ISG15, Viperin, and CXCL10) (**B**) were determined by qRT-PCR 48 h post-transfection (h.p.t.) based on two independent assays with three repeats each. (**C and D**) NC-M or miR-26a-M were transfected into HEK293T cells for 36 h, followed by VSV infection (MOI = 1) for 12 h. The relative mRNA levels of IFN-β (**C**) and representative ISGs (ISG15, Viperin, and CXCL10) (**D**) were detected by qRT-PCR based on two independent assays with three repeats each. (**E**) NC-M or miR-26a-M were co-transfected with IFN-β-Luc and pRL-TK plasmids into HEK293T cells for 36 h, followed by SeV infection (100 HAU/mL) for 12 h. The relative IFN-β promoter activities were measured by dual-luciferase reporter assay. (**F and G**) NC-M or miR-26a-M were transfected into HEK293T cells for 36 h, followed by SeV infection (100 HAU/mL) for 12 h. The relative mRNA levels of IFN-β (**F**) and ISGs (ISG15, Viperin, and CXCL10) (**G**) were determined by qRT-PCR based on two independent assays with three repeats each. Data are means ± SEM. Significance was calculated using two-tailed Student’s t test. *P < 0.05, **P < 0.01, and ***P < 0.001.

Generally, miRNAs exert their functions mainly in two ways, that is, interacting directly with RNA virus genomes or host genes. To investigate the antiviral mode-of-action of miR-26a, we first searched the possible targets of miR-26a on the HEV genome. Although the binding sites of miR-122 (served as a positive control) were successfully located on the HEV genome (19), no potential binding sites of miR-26a were discovered (Fig. S4). Intriguingly, in contrast to the potent antiviral effect observed in HepG2 cells, miR-26a exerted no antiviral effects on the innate immune defect Huh7 cells (Fig. S5A through C). Huh7 is a kind of innate immunodeficient cell line (20 – 23). The lack of antiviral effect of miR-26a on Huh7 cells prompted us to investigate whether the antiviral roles of miR-26a were achieved by the modulation of innate immune response. Indeed, we found that the transfection of miR-26a efficiently stimulated the expression of IFN-β (Fig. 2A) and the representative ISGs (RIG-I, ISG15, Viperin, and CXCL10) (Fig. 2B) in the HepG2-HEV-P6 model. In contrast, the expression of β2-Microglobulin (B2M) and phosphoglycerate kinase 1 (PGK1) was not affected (Fig. S6). These two genes are not innate immune-related genes, highlighting the specificity of miR-26a on type I IFN response. Consistently, miR-26a promoted the expression of IFN-β and the representative ISGs in the setting of VSV (Fig. 2C and D) or SeV infection (Fig. 2E through G). Taken together, our results demonstrated that miR-26a potently enhanced type I IFN responses to exert its broad antiviral effect.

### miR-26a specifically targets 3′UTR of mRNA to inhibit USP15 expression

**Fig 3:**
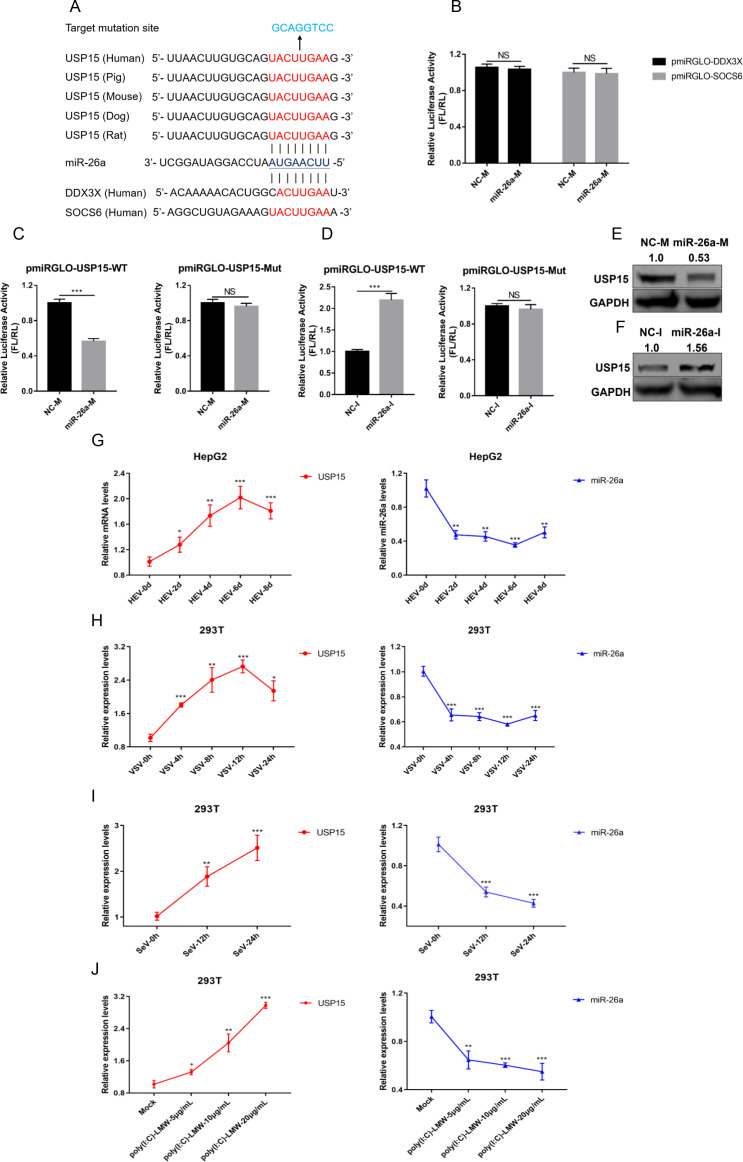
miR-26a targets the 3′UTR of USP15 to inhibit its expression. (**A**) Predicted target sites of miR-26a in the 3′UTR of USP15, DDX3X, and SOCS6 were illustrated. (**B**) NC-M or miR-26a-M were co-transfected with the corresponding 3′UTR reporter plasmids of DDX3X and SOCS5 into HEK293T cells, respectively. The relative luciferase activities were measured based on two independent assays with three repeats each. (**C**) NC-M or miR-26a-M were co-transfected with wild-type reporter plasmid (pmiRGLO-USP15-WT) or mutant reporter plasmid (pmiRGLO-USP15-Mut) into HEK293T cells, respectively. The relative luciferase activities were measured based on two independent assays with three repeats each. (**D**) Same as **C** for the transfection of NC-I or miR-26a-I. (**E**) NC-M or miR-26a-M were transfected into HepG2 cells for 48 h. The level of cellular USP15 was immunoblotted by WB. (**F**) Same as **E** for the transfection of NC-I or miR-26a-I. (**G**) The mRNA levels of USP15 were determined by qRT-PCR in HepG2 cells after being infected with HEV (60 copy number/cell) at indicated time points. The expression levels of USP15 (left panel) and miR-26a (right panel) were shown based on two independent assays with three repeats each. (**H–J**) HEK293T cells were infected with VSV (MOI = 1) (**H**) or SeV (100 HAU/mL) (**I**) at indicated time points, or HEK293T cells were transfected with poly(I:C)-LMW at different concentrations for 24 h (**J**). Then, the expression levels of USP15 (left panel) and miR-26a (right panel) were shown based on two independent assays with three repeats each. Data are means ± SEM. Significance was calculated using two-tailed Student’s t test. NS, no significance, *P < 0.05, **P < 0.01, and ***P < 0.001.

To further explore the antiviral mode-of-action of miR-26a, a miRNA target prediction program TargetScan (https://www.targetscan.org/vert_80/) was employed. Among the possible targets, we focused on USP15 (24), DEAD-box helicase 3 X-linked (DDX3X) (25), and suppressor of cytokine signaling 6 (SOCS6) (26), which may be the potential regulators of the type I IFN pathway. Their corresponding 3′ untranslated regions (3′UTRs) of mRNA were complementary to the miR-26a seed region (Fig. 3A). Therefore, wild-type or mutant 3′UTRs (the mutant region of 3′UTR was shown in Fig. 3A) were cloned into the pmiRGLO vector and coupled with the luciferase reporter gene, respectively. As shown in Fig. 3B, the transfection of miR-26a exerted no effect on the luciferase activities of DDX3X and SOCS6. Notably, overexpression of miR-26a significantly decreased 3′UTR-related luciferase activities of wild-type USP15, while having no effect on its mutant version (Fig. 3C). Conversely, knockdown of endogenous miR-26a by its inhibitors increased 3′UTR-related luciferase activities of wild-type USP15 but not its mutant version (Fig. 3D). Consistently, overexpression of miR-26a decreased the protein levels of USP15 (Fig. 3E). Moreover, knockdown of miR-26a leads to the accumulation of cellular USP15 (Fig. 3F). Collectively, we demonstrated that miR-26a targeted 3′UTR of USP15 mRNA to inhibit USP15 expression.

To further explore the relationship between miR-26a and USP15, the mRNA levels of both miR-26a and USP15 were detected in the presence of multiple viruses. As expected, in contrast with the expression of miR-26a, both HEV, VSV, and SeV infections or even poly (I:C)-LMW treatment promoted USP15 expression in both HEK293T (Fig. 3G through J) and HepG2 cells (Fig. S7). These observations further reinforce the fact that miR-26a targets 3′UTR of USP15 mRNA to inhibit the expression of USP15 protein.

### USP15 negatively regulates type I IFN signaling to promote virus replication

**Fig 4:**
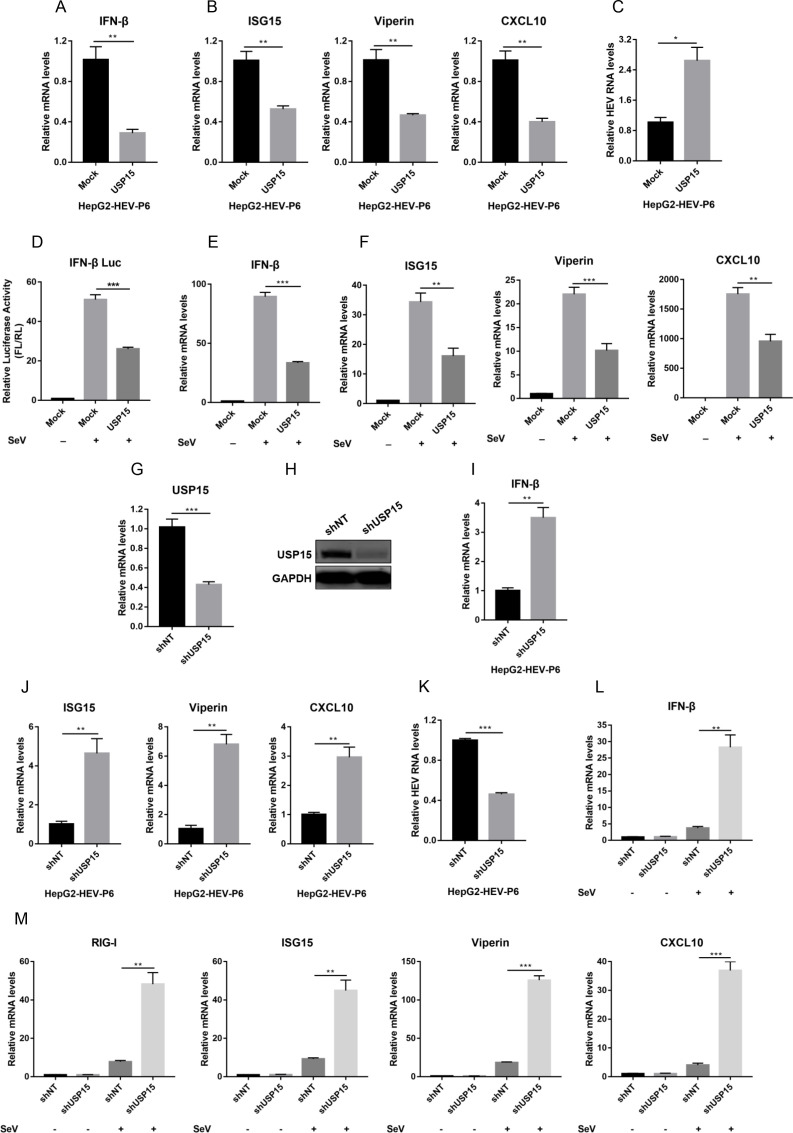
USP15 negatively regulates type I IFN signaling to promote HEV replication. (**A–C**) Empty vector (Mock) or HA-USP15 plasmid was transfected into HepG2-HEV-P6 cells for 24 h. The relative mRNA levels of IFN-β (**A**) and ISGs (RIG-I, ISG15, Viperin, and CXCL10) (**B**) or the relative levels of HEV RNA (**C**) were determined by qRT-PCR based on two independent assays with three repeats each. (**D**) Empty vector (Mock) or HA-USP15 plasmids were co-transfected with IFN-β-Luc and pRL-TK plasmids into HEK293T cells for 36 h, followed by SeV infection (100 HAU/mL) for 12 h. IFN-β promoter activity was determined based on two independent assays with three repeats each. (**E and F**) Empty vector (Mock) or HA-USP15 plasmids were transfected into HEK293T cells for 36 h, followed by SeV infection (100 HAU/mL) for 12 h. The relative mRNA levels of IFN-β (**E**) and ISGs (RIG-I, ISG15, Viperin, and CXCL10) (**F**) were determined by qRT-PCR based on two independent assays with three repeats each. (**G and H**) HEK293T cells stably expressing non-targeted shRNA (shNT) or shRNA targeting USP15 (shUSP15) were generated. The mRNA (**G**) or protein (**H**) levels of USP15 were evaluated by qRT-PCR or WB, respectively. (**I–K**) HepG2-HEV-P6 cells were infected with lentivirus expressing shNT or shUSP15 for 48 h. The relative mRNA levels of IFN-β (**I**) and ISGs (ISG15, Viperin, and CXCL10) (**J**) or the relative HEV RNA levels (**K**) were determined by qRT-PCR based on two independent assays with three repeats each, respectively. (**L and M**) HEK293T cells stably expressing shNT or shUSP15 were infected with SeV (100 HAU/mL) or mock infection for 12 h. The relative mRNA levels of IFN-β (**L**) and ISGs (RIG-I, ISG15, Viperin, and CXCL10) (**M**) were determined by qRT-PCR based on two independent assays with three repeats each. Data are means ± SEM. Significance was calculated using two-tailed Student’s t test. *P < 0.05, **P < 0.01, and ***P < 0.001.

USP15 was specifically targeted by miR-26a, we next investigated the roles of USP15 in the regulation of type I IFN response. In contrast to miR-26a, overexpression of USP15 suppressed the expression of IFN-β (Fig. 4A) and the representative ISGs (Fig. 4B), thus promoting HEV replication (Fig. 4C). Similarly, overexpression of USP15 also repressed the type I IFN responses (Fig. 4D and E) and ISG expression (Fig. 4F) in the presence of SeV. To further verify the role of USP15, we employed shRNA to stably knockdown USP15 expression (Fig. 4G and H). As expected, knockdown of USP15 enhanced the type I IFN responses (Fig. 4I) and ISG expression (Fig. 4J), thereby inhibiting HEV replication (Fig. 4K). Moreover, we observed similar results in the setting of SeV infection (Fig. 4L and M). Taken together, we showed that in contrast to miR-26a, USP15 negatively regulated the type I IFN response to facilitate virus replication.

### USP15 interacts directly with RIG-I to remove K63-linked ubiquitination of RIG-I

**Fig 5:**
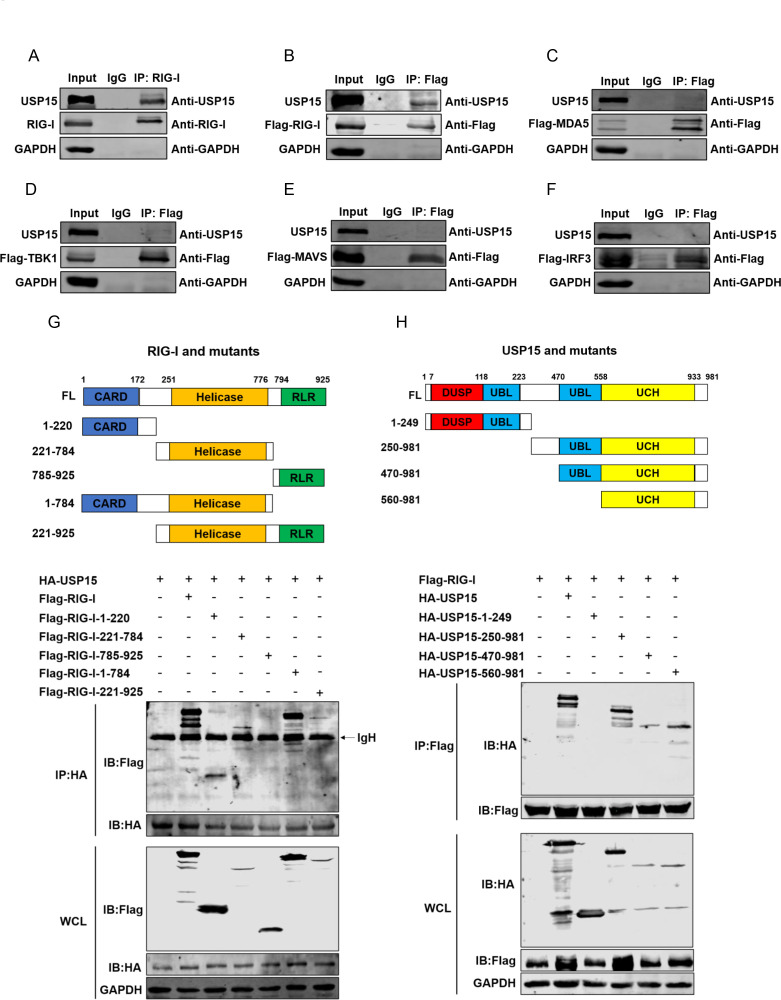
USP15 interacts directly with RIG-I. (**A**) HepG2 cells were lysed, followed by immunoprecipitation (IP) with anti-RIG-I or IgG antibodies; then, endogenous RIG-I and USP15 were detected by WB using anti-RIG-I and anti-USP15 antibodies. (**B–F**) Flag-tagged RIG-I (**B**), MDA5 (**C**), MAVS (**D**), TBK1 (**E**), and IRF3 (**F**) were transfected into HepG2 cells for 48 h, respectively. Then, the cells were lysed and whole-cell lysates (WCLs) were subjected to IP with anti-Flag or IgG antibodies. RIG-I and USP15 were immunoblotted using anti-Flag and anti-USP15 antibodies. (**G and H**) The constructs expressing the different domains of RIG-I (**G**) and USP15 (**H**) were illustrated. Various constructs were transfected into HEK293T cells for 48 h and subjected to IP with anti-Flag or anti-HA antibodies, followed by immunoblotting (IB) analysis using anti-Flag or anti-HA antibodies. IgH represents the heavy chain of antibody.

Aiming to dissect the mode-of-action of USP15 in type I IFN responses, we performed co-immunoprecipitation (co-IP) assays to check the possible USP15-interacting elements within IFN pathway (e.g., RIG-I, MDA5, TBK1, MAVS, and IRF3). Specifically, we found that USP15 interacted with both endogenous and exogenous RIG-I (Fig. 5A and B), whereas other elements showed no interaction (Fig. 5C through F). To further delineate the key interaction domains between USP15 and RIG-I, a series of truncated mutants was constructed and subjected to co-IP assay accordingly (Fig. 5G and H). We found that two domains of RIG-I, the N-terminal tandem CARD domain and the middle helicase domain, interact directly with USP15 (Fig. 5G). And the C-terminal UCH domain of USP15 was essential for the interaction with RIG-I (Fig. 5H).

**Fig 6:**
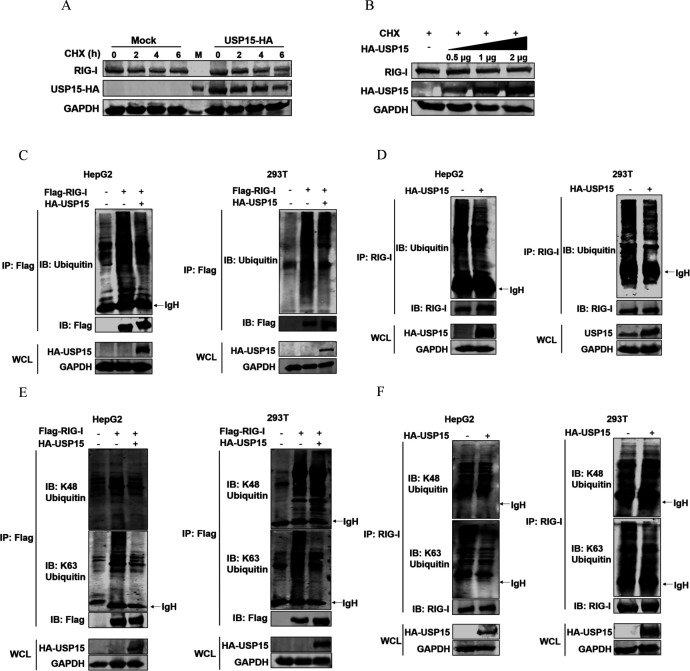
USP15 removes K63-lined ubiquitination from RIG-I. (**A**) Empty vector (Mock) or HA-tagged USP15 were transfected into HepG2 cells, followed by treatment with 30 µg/mL CHX for indicated times. The protein levels of endogenous RIG-I were measured by WB. (**B**) Different doses of HA-tagged USP15 were transfected into HepG2 cells, followed by treatment with CHX for 6 h. The protein levels of endogenous RIG-I were measured by WB. (**C and E**) Flag-tagged RIG-I and HA-tagged USP15 were co-transfected into HepG2 or HEK293T cells for 48 h. Cells were lysed and immunoprecipitated with anti-Flag antibody. Then, the levels of endogenous ubiquitinated RIG-I were detected by WB using anti-Ub (**C**) or anti-K48-Ub and anti-K63-Ub antibodies (**E**). (**D and F**) Empty vector (Mock) or HA-tagged USP15 were transfected into HepG2 or HEK293T cells for 48 h. Cells were lysed and subjected to immunoprecipitation with anti-RIG-I antibody. The levels of endogenous ubiquitination of RIG-I were detected by WB using anti-Ub (**D**) or anti-K48-Ub and anti-K63-Ub antibodies (**F**), respectively. IgH represents the heavy chain of antibody.

To investigate the possible consequences of protein-protein interaction on RIG-I, HA-USP15 was transfected into HepG2 cells with the presence of cycloheximide (CHX). WB analysis showed that overexpression of USP15 exerted no significant effect on the stability of RIG-I (Fig. 6A and B). USP15 is a member of deubiquitinates. It could remove the ubiquitin from substrate proteins to regulate protein function (27). Therefore, we measured the levels of ubiquitinated RIG-I with the overexpression of USP15. Importantly, overexpression of USP15 decreased the levels of ubiquitinated RIG-I both exogenously and endogenously in HepG2 and HEK293T cells (Fig. 6C and D). Two forms of RIG-I ubiquitination have been characterized. The ubiquitin E3 ligases TRIM25 or Riplet attaches K63-linked polyubiquitin chains to RIG-I to promote the activation of the RIG-I pathway (18), whereas E3 ligase, RNF125, conjugates K48-linked ubiquitin to RIG-I to inhibit RIG-I activation (28). Thus, the levels of ubiquitinated RIG-I were determined with both anti-K48-Ub and anti-K63-Ub antibodies. Remarkably, USP15 removed the K63-linked ubiquitination of RIG-I both exogenously and endogenously, while exerting no significant effect on the K48-linked ubiquitination (Fig. 6E and F). Collectively, we demonstrated that USP15 interacts directly with RIG-I to deubiquitinate K63-linked RIG-I.

### miR-26a, by downregulating USP15, promotes the K63-linked ubiquitination of RIG-I to enhance type I IFN responses

**Fig 7:**
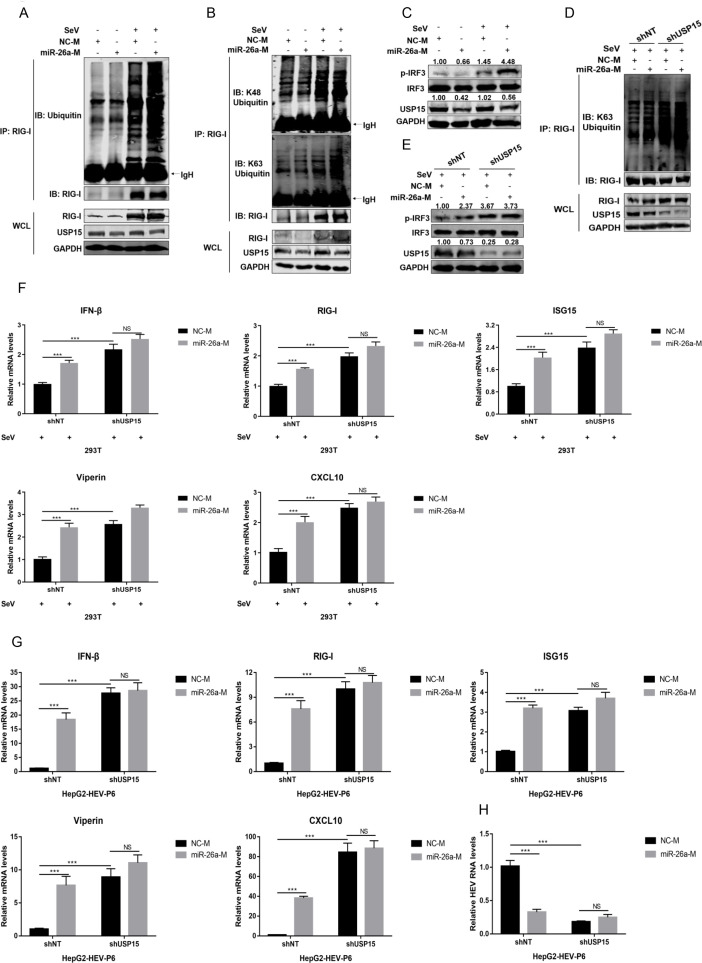
miR-26a, by downregulating USP15, promotes the K63-linked ubiquitination of RIG-I to enhance type I IFN responses. (**A and B**) NC-M or miR-26a-M were transfected into HEK293T cells for 36 h, followed with or without SeV infection (100 HAU/mL) for 16 h. Then, cells were lysed and subjected to immunoprecipitation with anti-RIG-I antibody. The endogenous ubiquitinated RIG-I was analyzed by immunoblots using anti-Ub (**A**) or anti-K48-Ub and anti-K63-Ub (**B**) antibodies, respectively. IgH represents the heavy chain of antibody. (**C**) NC-M or miR-26a-M were transfected into HEK293T cells for 36 h, followed with or without SeV infection (100 HAU/mL) for 16 h. The level of phosphorylated IRF3 (p-IRF3) was detected by WB using anti-phospho-IRF3 (Ser396) antibody. (**D and E**) NC-M and miR-26a-M were transfected into 293T-shNT or 293T-shUSP15 cells for 36 h, followed by SeV infection (100 HAU/mL) for 16 h. Then, the cells were lysed and subjected to immunoprecipitation with anti-RIG-I antibody, and the endogenous ubiquitinated RIG-I was immunoblotted using anti-K63-Ub antibody (**D**). The level of p-IRF3 was detected using anti-phospho-IRF3(Ser396) antibody (**E**). (**F**) NC-M and miR-26a-M were transfected into 293T-shNT or 293T-shUSP15 cells for 36 h, followed by SeV infection (100 HAU/mL) for 16 h. The mRNA levels of IFN-β and ISGs (RIG-I, ISG15, Viperin, and CXCL10) were detected by qRT-PCR based on two independent assays with three repeats each. (**G and H**) NC and miR-26a mimics were transfected into HepG2-HEV-P6 cells for 24 h, followed by infection with lentivirus expressing shNT or shUSP15 for 48 h. Then, the mRNA levels of IFN-β and ISGs (RIG-I, ISG15, Viperin, and CXCL10) (**G**) or the relative levels of HEV RNA (**H**) were determined by qRT-PCR based on two independent assays with three repeats each, respectively. Data are means ± SEM. Significance was calculated using two-tailed Student’s t test.NS, no significance, ***P < 0.001.

**Fig 8:**
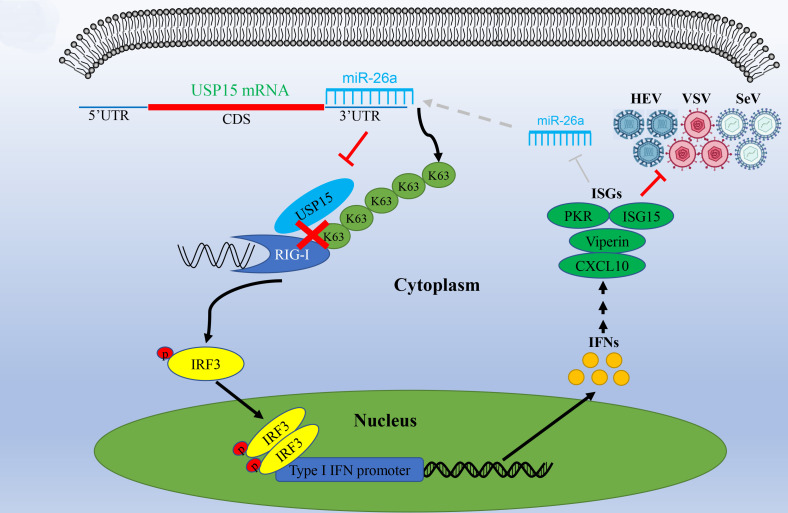
miR-26a enhances RIG-I-mediated type I IFN response by targeting USP15 to suppress viral replications. USP15 interacts with RIG-I to remove K63-lined ubiquitination. miR-26a targets the 3′UTR of mRNA to inhibit USP15 expression. Consequently, miR-26a promoted K63-linked ubiquitination of RIG-I to enhance type I IFN antiviral response, suppressing HEV, VSV, and SeV replication. Being an intricate regulatory network, the activation of type I IFN responses could suppress miR-26a expression to avoid the disordered activation that might result in the so-called “type I interferonopathy.” Black lines represent “promoting,” red lines represent “inhibiting,” and gray dotted lines represent “negative feedback.”

miR-26a inhibits USP15 expression, while USP15 removed K63-linked ubiquitination of RIG-I. Therefore, we want to investigate whether miR-26a could regulate RIG-I and the related IFN responses. miR-26a was transfected into HepG2 cells, followed by SeV infection. Our results indicated that miR-26a significantly enhanced the levels of ubiquitinated RIG-I (Fig. 7A, right panel), specifically the K63-linked but not K48-linked ubiquitination (Fig. 7B, right panel). Consistent with the pro-activation role of K63-lined RIG-I, miR-26a also promoted IRF3 phosphorylation (Fig. 7C). Moreover, knockdown of USP15 increased the levels of K63-lined ubiquitinated RIG-I (Fig. 7D, lane 1 and lane 3) and phosphorylated IRF-3 (Fig. 8E, lane 1 and lane 3), whereas miR-26a induced K63-RIG-I and phosphorylated IRF-3 was largely abrogated in the setting of USP15 knockdown (Fig. 7D and E, lane 3 and lane 4). Correspondingly, USP15 knockdown stimulated the expression of IFN-β and ISGs in the presence of SeV (Fig. 7F, lane 1 and lane 3) or HEV (Fig. 7G, lane 1 and lane 3), whereas miR-26a failed to achieve further stimulation once USP15 was downregulated (Fig. 7F and G, lane 3 and lane 4). Consequently, knockdown of USP15 efficiently blocked HEV replication (Fig. 7H, lane 1 and lane 3). And miR-26a potently inhibited HEV replication in control cells (Fig. 7H, lane 1 and lane 2) but not in USP15 knockdown cells (Fig. 7H, lane 3 and lane 4). Collectively, we concluded that miR-26a, by downregulating USP15, promotes the activation of the RIG-I pathway, resulting in an active IFN response to suppress viral replication.

## DISCUSSION

Currently, the most commonly used antiviral strategies are vaccine prevention and drug treatments. However, due to the rapid evolution and drug resistance of viruses, there remains an urgent need to develop new and effective broad-spectrum antiviral strategies. IFN-I is eﬀective against different viruses through various mechanisms. Herein, we identified a novel host factor involved in innate immune against viral infection, miR-26a.

miRNAs are small (up to 25 nt), non-coding highly conserved RNAs, which represent 1% of the human genome but interact with about 60% of messenger RNAs. The matured mi-RNAs recognize their complementary mRNAs through base-pairing, which occurs between the so-called miRNA’s “seed region” (located on the 2nd to 7th nt of 5′-end) and the miRNA binding site within 3′UTR (29). Host miRNAs are essentially involved in the life cycles of multiple viruses and their associated pathogenesis via interacting directly with viral genomes or host genes. For instance, miR-122 and miR-214 facilitate HEV replication via direct binding to the target sites in the viral genome (19, 30). miR-146 facilitates dengue virus replication by impaired IFN-I production by targeting TRAF6 (31); miR-526 also enhanced IFN-I production by targeting CYLD to inhibit EV71 replication (32). Besides their location in tissues or cells, miRNAs also circulate in serum and other extracellular bio-fluids (e.g., cerebrospinal fluid, saliva, and urine). Thus, specific miRNA signatures could be explored as diagnostic or prognostic biomarkers and help in the development of new therapeutic interventions (33). However, due to the lack of in-depth understanding between miRNAs and viral life cycles, the development of miRNA-based antiviral strategies is impeded.

Although a previous study has also reported that miR-26a targets USP3 to enhanced type I IFN signaling, the specific regulatory mechanism of miR-26a has not been clearly clarified (34). Herein, we demonstrated that miR-26a, by downregulating USP15, promotes RIG-I K63-ubiquitination to enhance type I IFN antiviral responses. The ability to harness the body’s own immune system to fight virus infection continues to be a key research line for the development of antivirals. For instance, numerous small molecule agonists of Toll-like receptors 7 and 8 have been developed and evaluated in clinical trials for combating chronic hepatitis virus infection (35, 36). Along a similar research line, our study showed that miR-26a serves as a potent positive regulator of IFN responses, highlighting the potential that miR-26a or its mimics can be further explored as a broad antiviral agent.

On the other hand, in our study, we found that USP15 suppressed the type I interferon signaling by removing K63-linked of RIG-I ubiquitination, which is consistent with one previous research (24) but contrary with the results described in Pauli’s paper (37). Moreover, another study also demonstrated that USP15 was recruited by UBE2S to remove K63-linked ubiquitination of TBK1 to inhibit type I IFN production (38). We speculate that USP15 may regulate type I IFN antiviral signaling through different mechanisms.

miRNA-based antiviral therapeutics are evolving and represent a promising therapeutic option. For example, miravirsen, developed by Santaris Pharma A/S, was the first miRNA-based drug that successfully completed a phase 2 clinical trial for the treatment of chronic hepatitis C virus (HCV) infection (39). Serving as a miR-122 antagonist, miravirsen reduces HCV RNA levels by sequestering miR-122 away from the viral genome, where it is needed to enhance HCV propagation (40). In addition, this study provided new insights into how host microRNA fights against viral infection. Therefore, more investigations into interactions between miRNAs and viruses are highly needed for the development of miRNA-based antiviral therapies against viruses. Therefore, miR-26a may have the potential to become a novel drug target in virally induced diseases.

In our study, we also found that the intense activation of type I IFN responses could suppress miR-26a expression in turn. This observation highlights the importance of intrinsic negative feedback loops within the innate immune responses, ensuring a balanced activation to avoid the so-called “type I interferonopathy” (41).

## MATERIALS AND METHODS

### Cell culture, plasmids, and antibodies

Huh7, HepG2, and HEK293T cells were cultured in Dulbecco’s modified Eagle medium (Bio-Channel, China) supplemented with 10% fetal bovine serum (Bio-Channel, China) and penicillin-streptomycin. Flag-tagged RIG-I, MDA5, MAVS, TBK1, IRF3, HA-tagged WT-Ub, K48-Ub, and K63-Ub plasmids were kept in our laboratory. HA-tagged USP15 were created by cloning the CDS (coding sequence) region of USP15 into the pcDNA3.1-HA vector. Plasmids, miRNA mimics, and inhibitors were transfected into cells using M5 Hiper Lip.2000 (Mei5bio) according to the manufacturer’s instructions. All the antibodies used in this research were listed as follows: anti-Flag (M20008) and anti-HA (M20003) antibodies were purchased from Abmart; anti-RIG-I (20566–1-AP), anti-USP15 (14354–1-AP), anti-Ub (10201–2-AP), and anti-GAPDH (60004–1-Ig) were purchased from Proteintech; anti-K48-Ub (8081), anti-K63-Ub (5621), and anti-Phospho-IRF3 (37829) were obtained from Cell Signaling Technology.

### miRNA mimics and inhibitors

miR-26a mimics (double-stranded RNA), inhibitors (single-stranded RNA), and their corresponding controls were synthesized by GenePharma. The sequences were listed as follows: NC mimics, 5′-UUCUCCGAACGUGUCACGUTT-3′ (sense), and 5′-ACGUGACACGUUCGGAGAATT-3′ (anti-sense); miR-26a mimics, 5′-UUCAAGUAAUCCAGGAUAGGCU-3′ (sense) and 5′-CCUAUCCUGGAUUACUUGAAUU-3′ (antisense); NC inhibitors, 5′-CAGUACUUUUGUGUAGUACAA-3′; miR-26a inhibitors: 5′-AGCCUAUCCUGGAUUACUUGAA-3′.

### *In vitro* RNA synthesis

The plasmid containing the full-length HEV genome (Kernow-C1 P6 clone, GenBank Accession Number: JQ679013) was linearized by MluI as described previously (42). Subsequently, full-length HEV RNA was transcribed and capped *in vitro* by the Ambion mMESSAGE mMACHINE RNA transcription Kit (Thermo Fisher, AM1344).

### Construction of HEV cell culture models

HEV RNA synthesized *in vitro* was delivered into Huh7 or HepG2 cells using BTX ECM630 electroporation systems as described previously, named Huh7-HEV-P6 or HepG2-HEV-P6, respectively. Briefly, 10^7^ cells were washed with PBS and resuspended by 400 µL Opti-MEM and mixed with 10 µg full-length HEV RNA transcripts. Electroporation parameters were followed: pulsing once with 975 µF and 270 V, time constants between 18 and 20 msec. Consequently, HEV can replicate persistently even after passaging for several generations (43).

### Luciferase reporter assays

#### 3′UTR luciferase reporter assays

The 3′UTR sequences of human USP15, DDX3X, and SOCS6 were amplified and cloned into the pmiRGLO vector (a vector expressing both firefly and Renilla luciferase), respectively. The 3′UTR of USP15 complementary to the miR-26a seed region were mutated and cloned into the pmiRGLO vector as well, named pmiRGLO-USP15-Mut. Then, the wild-type or mutant plasmids were co-transfected with miR-26a mimics or inhibitors. Thirty-six hours post-transfection, the luciferase values were measured using the duo-lite luciferase reporter assay system (Vazyme, DL101-01) according to the manufacturer’s instructions. Data were normalized by determining the ratios of firefly luciferase activities to that of Renilla luciferase.

#### IFN-β luciferase reporter assays

Two hundred fifty nanograms IFN-β-Luc plasmid (containing IFN-β luciferase promoter) was co-transfected with 50 ng pRL-TK (a plasmid expressing the Renilla luciferase protein was used as an internal control) into HEK293T cells. Luciferase activity was measured as described above.

### Indirect immunofluorescence

Briefly, cells were fixed with 4% paraformaldehyde for 30 min, followed by permeabilization with 0.3% Triton X-100 for 15 min at room temperature. Then, the cells were incubated with rabbit anti-ORF2 polyclonal antibody (1:2,000) overnight at 4°C, followed by incubation with CoraLite594-conjugated goat anti-rabbit IgG(H+L) (Proteintech, SA00013-4, 1:500) at 37°C for 1 h. Finally, cellular nuclei were stained with Hoechst 33342 (Thermo Fisher, H1399, 1:500) for 10 min at room temperature. After washing three times with PBST buffer, cells were visualized using an ﬂorescence microscope (Olympus).

### Quantitative reverse transcription PCR

Total RNA was extracted with TRIzol reagent (Ambion) according to the manufacturer’s instructions. For the quantification of miR-26a, RNA was reverse transcribed with miR-26a stem-loop RT primer and U6 reverse primer using the miRNA First-Strand cDNA Synthesis Kit (Vazyme, MR101-01). For cellular gene quantification, such as IFN-β, ISG15, Viperin, CXCL10, and RIG-I, RNA was reverse transcribed using HiScript Q RT SuperMix for quantitative real-time PCR (qPCR) (Vazyme, R122-01). qPCR was performed using LightCycler 480 system (Roche) with the condition of 95°C for 2 min, followed by 40 cycles of 95°C for 30 s, 60°C for 30 s, and 72°C for 30 s. And relative expression levels of miRNAs or mRNAs were normalized to that of U6 or β-actin via the 2^−△△^ *^CT^* threshold method, respectively. All the primers used were listed in Table S1.

### Co-immunoprecipitation and immunoblot analyses

HEK293T cells were transfected with various expression plasmids. Forty-eight hours after transfection, cells were harvested and lysed by Nonidet P-40 lysis buffer (Beyotime, ST2045). The whole-cell lysates were centrifuged at 12,000 rpm for 5 min at 4°C. Then, the supernatants were incubated and immunoprecipitated with 0.5 µg antibodies overnight at 4°C, followed by incubation with 30 µL magnetic bead for 8 h at 4°C. Then, the beads were washed with PBS buffer three to five times. The bound proteins were separated by SDS-PAGE and analyzed by immunoblots.

### Construction of the USP15 knockdown HepG2 cell line

The lentiviral pseudo particles expressing non-targeting control shRNA or USP15-specific shRNA were generated in HEK293T cells using the lentivirus packaging system as described before (22). The shRNA sequences targeting USP15 were listed in Table S2. The lentivirus was collected and stored at −80°C. HepG2 cells were seeded into 6-well plates at a density of 3 × 10^5^ per well and transduced with lentiviral pseudo particles supplemented with polybrene (1:2,000) at 37°C for 2 days. To obtain the USP15 stable knockdown cell line, cells transduced with shRNA lentivirus were selected by puromycin at a concentration of 2.5 µg/mL. Then, the effect of knockdown was evaluated by qRT-PCR and WB.

### *In vitro* ubiquitination assay

Cells were treated with 20 µM MG132 (MedChemExpress, MCE, HY-13259) for 4 h, followed by lysis with Nonidet P-40 lysis buffer supplemented with protease inhibitor cocktail and proteasome inhibitor MG132. After the sample was sonicated, the WCL was immunoprecipitated with anti-Flag or anti-RIG-I antibodies overnight and then incubated with the magnetic bead (MCE, HY-K0202) for 8 h. The endogenous ubiquitinated RIG-I was analyzed by immunoblots using anti-Ub, anti-K48, or anti-K63 antibodies, while exogenous ubiquitinated RIG-I was detected by the anti-HA antibody.

### Statistical analysis

All the data are representative of two or three independent experiments. The significant differences were calculated using two-tailed Student’s t test (NS > 0.05, *P < 0.05, **P < 0.01, and ***P < 0.001).

## SUPPLEMENTAL MATERIAL

Supplemental material spectrum.03124-23-s0001.pdf Fig. S1 to S7 and Tables S1 and S2.

ASM does not own the copyrights to Supplemental Material that may be linked to, or accessed through, an article. The authors have granted ASM a non-exclusive, world-wide license to publish the Supplemental Material files. Please contact the corresponding author directly for reuse.
