# Supplementary material for: miR-26a exerts broad-spectrum antiviral effects via the enhancement of RIG-I-mediated type I interferon response by targeting USP15
Source: Microbiol Spectr. 2023 Nov 29;12(1):e03124-23. doi: 10.1128/spectrum.03124-23 (PMC10783007; doi:10.1128/spectrum.03124-23)
Supplement: Supplemental material — Fig. S1 to S7 and Tables S1 and S2. [file spectrum.03124-23-s0001.pdf]

Supplementary data

miR-26a exerts broad-spectrum antiviral effects via the enhancement of RIG-I-mediated type I Interferon response by targeting USP15

Jikai Zhang[1,2], Chunyang Li[1,2], Yao Hou[1,2], Dan Liu [1,2], Qiudi Li[1,2], Zijie Wang[1,2], Renxian Tang[1,2], Kuiyang Zheng[1,2], Hongbo Guo[1,2*], Wenshi Wang[1,2*]

[1]Jiangsu Key Laboratory of Immunity and Metabolism, Department of Pathogenic Biology and Immunology, Xuzhou Medical University, Xuzhou, China.

[2]Jiangsu International Laboratory of Immunity and Metabolism, Xuzhou Medical University, Xuzhou, China.

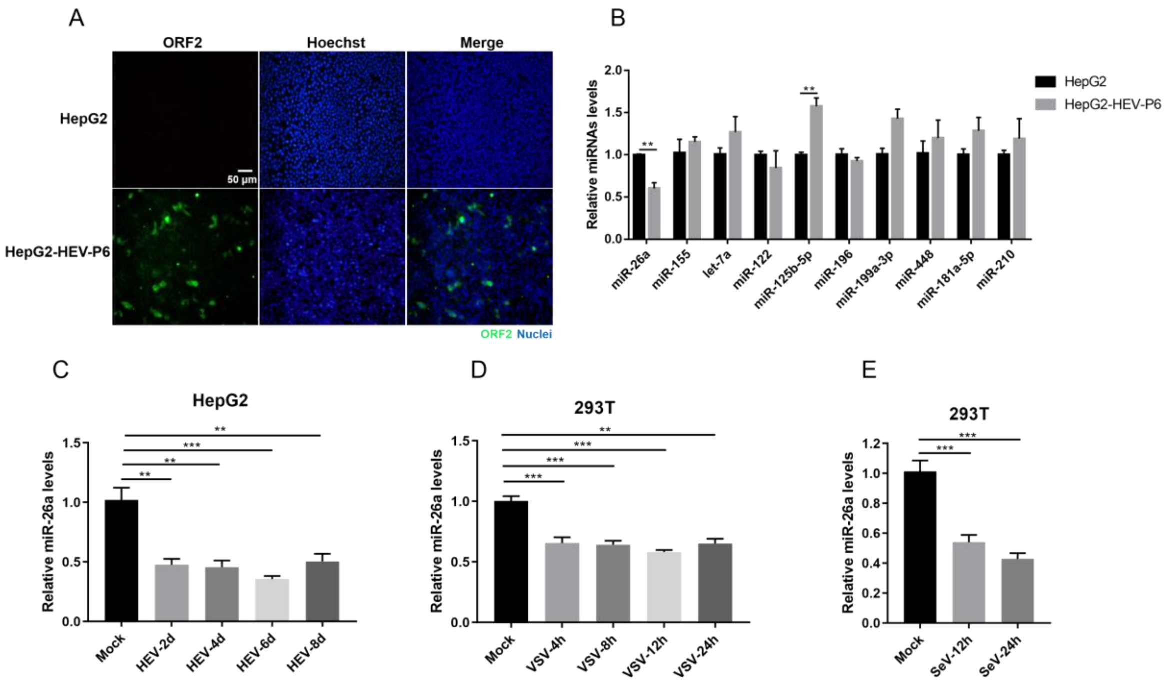

FIG S1 HEV, VSV and SeV infection downregulated miR-26a expression levels. (A) HepG2-HEV-P6 model was constructed by the delivery of the full-length HEV genome RNA into HepG2 cells. After three times of passages, ORF2 protein can still be successfully detected by IFA, indicating continuous HEV replication in this cell model. **(B)** The expression of various miRNAs was determined by stem-loop qRT-PCR in HepG2 or HepG2-HEV-P6 cells. **(C)** HepG2 cells were infected with HEV (60 copy number/cell). The cellular miR-26a expression levels were determined by qRT-PCR and normalized to the expression of U6 based on two independent assays with three repeats each. **(D and E)** HEK293T cells were infected with VSV (MOI=1) **(D)** or SeV (100 HAU/mL) **(E)** for the indicated times. The cellular miR-26a expression levels were determined by qRT-PCR and normalized to the expression of U6 based on two independent assays with three repeats each. Data are means±SEM. Significance was calculated using two-tailed Student's t test. ** $P < 0.01$, *** $P < 0.001$. IFA: indirect immunofluorescence assay; MOI: multiplicity of infection; HAU: hemagglutinating units.

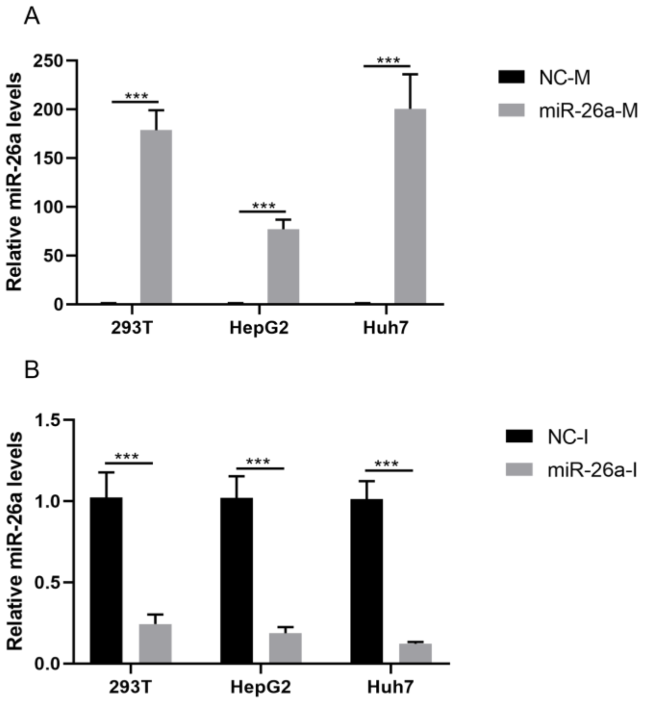

FIG S2 Validation of the transfection efficacy of miR-26a mimics and inhibitors. NC, miR-26a mimics **(A)** or NC, miR-26a inhibitors **(B)** were transfected into HEK293T, HepG2 or Huh7 cell, respectively. Then the levels of miR-26a were determined by qRT-PCR. Data are means±SEM. Significance was calculated using two-tailed Student's t test. *** $P < 0.001$.

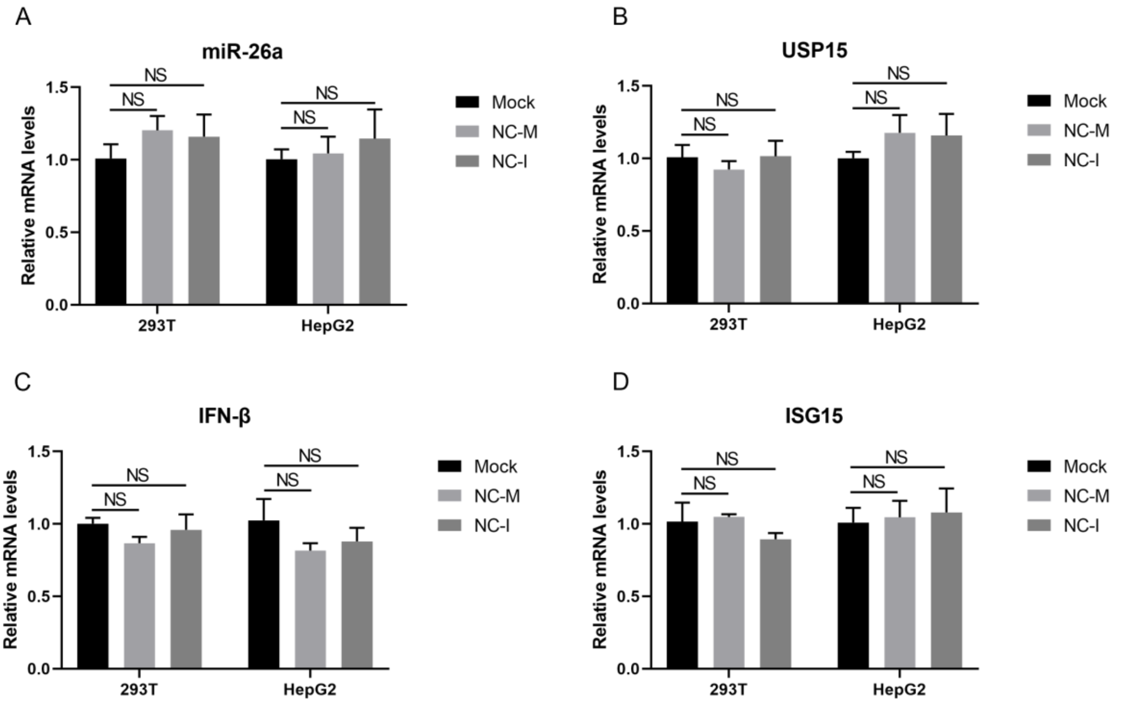

FIG S3 Assessment of any possible nonspecific effects of miRNA transfection. NC mimics or inhibitors were transfected into HepG2 or HEK293T cells, and cells without transfection served as Mock group. The mRNA expression levels of miR-26a **(A)**, USP15 **(B)**, IFN-β **(C)** and ISG15 **(D)** were determined by qRT-PCR, respectively. Data are means±SEM. Significance was calculated using two-tailed Student's t test. NS, no significance.

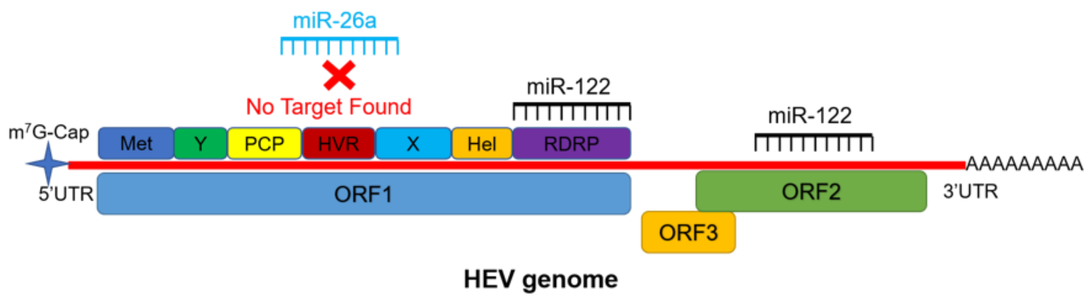

FIG S4 The predicted target sites of miR-26a and miR-122 (positive control) were illustrated on HEV genome.

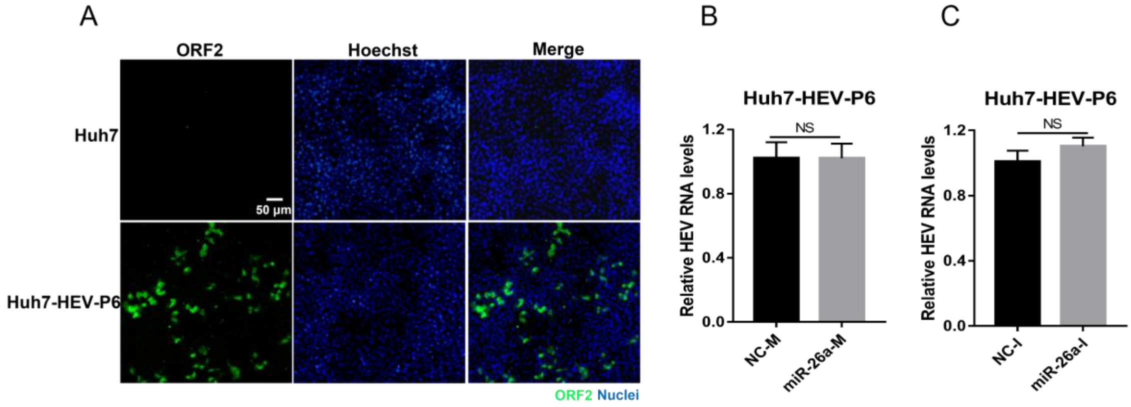

FIG S5 miR-26a has no effects on HEV replication in Huh7 cells. (A) Huh7-HEV-P6 replication model was constructed by the delivery of the full-length HEV genome RNA into Huh7 cells. After three times of passages, ORF2 protein can still be successfully detected by IFA, indicating continuous HEV replication in this cell model. **(B and C)** NC-M, miR-26a-M **(B)** or NC-I, miR-

26a-I **(C)** were transfected into Huh7-HEV-P6 cells, respectively. The relative levels of HEV RNA were determined by qRT-PCR 48 h.p.t. based on two independent assays with three repeats each. Data are means ± SEM. Significance was calculated using two-tailed Student's t test. NS, no significance.

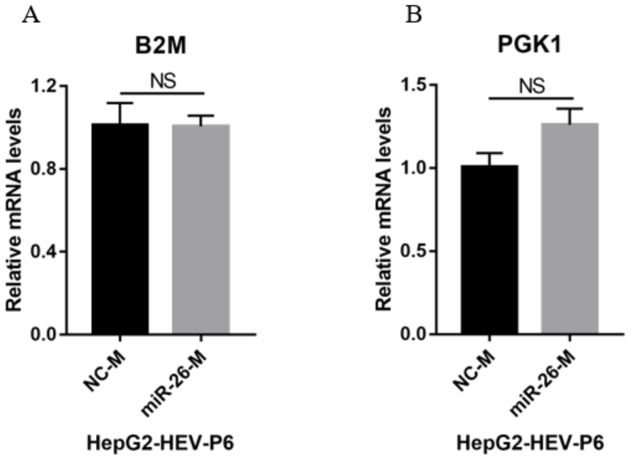

FIG S6 Overexpression of miR-26a exerts no effect on the expression of B2M and PGK1. NC-M and miR-26a-M were transfected into HepG2-HEV-P6 cells. The relative mRNA levels of B2M **(A)** and PGK1 **(B)** were determined by qRT-PCR 48 h.p.t. based on two independent assays with three repeats each. Data are means±SEM. Significance was calculated using two-tailed Student's t test. NS, no significance.

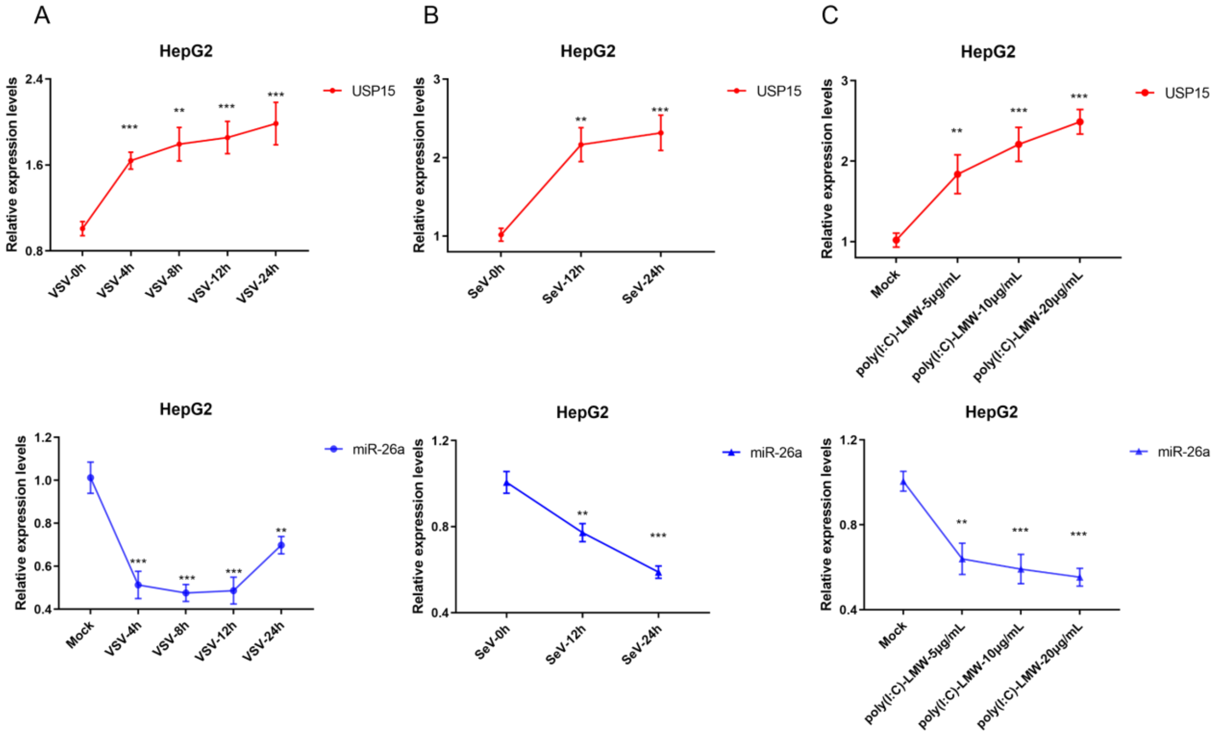

FIG S7 Virus infection or poly (I:C) treatment inhibited miR-26a expression and promoted USP15 expression in HepG2 cells. HepG2 cells were infected with VSV (MOI=1) **(A)** or SeV (100 HAU/mL) **(B)** for the indicated times, or HepG2 cells were transfected with poly (I:C)-LMW **(C)** at different concentrations for 24 h. Cellular USP15 or miR-26a expression levels were determined by qRT-PCR and normalized to the expression of U6 or GAPDH based on two independent assays with three repeats each. Data are means±SEM. Significance was calculated using two-tailed Student's t test. ** $P < 0.01$, *** $P < 0.001$.

Table S1. Primer sequences for qRT-PCR

Primers	Sequences (5’-3’)
RT-miR-26a	GTCGTATCCAGTGCAGGGTCCGAGGTATTCGCACTGGATAC GACAGCCTATC
miR-26a-F	CGGGGCTTCAAGTAATCCAG
miR-UR-R	CAGTGCAGGGTCCGAGGTAT
U6-F	CTCGCTTCGGCAGCACA
U6-R	AACGCTTCACGAATTTGCGT
qHEV-F	TTGCCTCCGAGTTAGTCATC
qHEV-R	TGCAAAGCATTACCAGACCG
qSeV-F	GACAGATGAGATATCGTGGATGG
qSeV-R	TCTAAGCTCAGATTAGCCCTTGT
qIFN-β-F	ATGACCAACAAGTGTCTCCTCC
qIFN-β-R	GCTCATGGAAAGAGCTGTAGTG
qISG15-F	CTCTGAGCATCCTGGTGAGGAA
qISG15-R	AAGGTCAGCCAGAACAGGTCGT
qViperin-F	CCAGTGCAACTACAAATGCGGC
qViperin-R	CGGTCTTGAAGAAATGGCTCTCC
qCXCL10-F	GGTGAGAAGAGATGTCTGAATCC
qCXCL10-R	GTCCATCCTTGGAAGCACTGCA
qRIG-I-F	CACCTCAGTTGCTGATGAAGGC
qRIG-I-R	GTCAGAAGGAAGCACTTGCTACC
qGAPDH-F	GTCTCCTCTGACTTCAACAGCG
qGAPDH-R	ACCACCCTGTTGCTGTAGCCAA
qUSP15-F	CGACGCTGCTCAAAACCTC
qUSP15-R	TCCCATCTGGTATTTGTCCCAA

Table S2. Lentiviral shRNA sequences

No.	Gene	Accession	Sequences	Target sequence
shUSP15	Ubiquitin Specific Peptidase 15	NM_001252078.2	TGCTGTTGACAGTGAGCGCACAGTCAGTCTGAAGATTCAGTAGTGAAGCCACAGATGTACTGAATCTTCAGACTGACTGTTTGCCTACTGCCTCGGA	AACAGTCAGTCTGAAGATTCAG
